# Municipality-level measles, mumps, and rubella (MMR) vaccine coverage and deprivation in Brazil: A nationwide ecological study, 2006 to 2020

**DOI:** 10.1371/journal.pgph.0002027

**Published:** 2023-08-01

**Authors:** Audrey Godin, Julia M. Pescarini, Amber I. Raja, Enny S. Paixao, Maria Yury Ichihara, Ana Paula S. Sato, Liam Smeeth, Mauricio L. Barreto, Elizabeth B. Brickley

**Affiliations:** 1 Health Equity Action Lab, Department of Infectious Disease Epidemiology, London School of Hygiene & Tropical Medicine, London, United Kingdom; 2 Centro de Integração de Dados e Conhecimentos para Saúde, Fundação Oswaldo Cruz, Salvador, Brasil; 3 Faculdade de Saúde Pública, Universidade de São Paulo, São Paulo, Brasil; 4 Department of Non-communicable Disease Epidemiology, London School of Hygiene & Tropical Medicine, London, United Kingdom; University of Embu, KENYA

## Abstract

To better understand the declining rates of routine childhood vaccination in Brazil, we investigated the association between measles, mumps, and rubella (MMR) first dose vaccine coverage and deprivation at the municipality level. Using routinely collected data from 5565 Brazilian municipalities from 2006 to 2020, we investigated the association between municipality-level MMR vaccine first dose coverage (i.e., as a continuous variable and as a percentage of municipalities attaining the 95% target coverage) in relation to quintiles of municipality-level deprivation, measured by the Brazilian Deprivation Index (*Índice Brasileiro de Privação*, IBP), and geographic regions. From 2006 to 2020, the mean municipality-level MMR vaccine coverage declined across all deprivation quintiles and regions of Brazil, by an average of 1.2% per year. The most deprived quintile of municipalities had higher coverage on average, but also the steepest declines in coverage (i.e., an annual decline of 1.64% versus 0.61% in the least deprived quintile) in the period of 2006–2020, and the largest drop in coverage at the beginning of the COVID-19 pandemic (2019–2020). Across all deprivation quintiles and regions (except for the Southeast region), less than 50% of municipalities in Brazil met the 95% MMR coverage target in 2020.The decrease in MMR first dose vaccine coverage in Brazil is widespread, but steeper declines have been observed in the most deprived municipalities. To promote vaccine equity and prevent future outbreaks, further research is urgently needed to understand the causal mechanisms underlying the observed associations between municipality-level MMR vaccine coverage and deprivation.

## Introduction

Routine childhood vaccination coverage has been plateauing, and even decreasing, in some regions of the world over the last decade [1]. Shortfalls in vaccine coverage may increase the burden of endemic infectious diseases and the risk of re-emergence for locally eliminated pathogens. The reasons for the decreases in coverage are complex and multifactorial but likely include a combination of growing vaccine hesitancy, lack of awareness of the risks associated with vaccine-preventable diseases, and increasingly complex vaccine schedules [2, 3].

In Brazil, the National Immunization Program (*Programa Nacional de Imunizações*, PNI) is tasked with providing and promoting free vaccines and has historically achieved high vaccination coverage, contributing to a significant reduction in the number of cases of vaccine-preventable diseases, such as measles [3]. In the first Brazilian National Vaccination Calendar published in 1977, the monovalent measles vaccine was one of the four mandatory vaccines administered in the first year of life. In 2003, the measles, mumps, and rubella (MMR) trivalent vaccine became the recommended measles-containing vaccine for children aged 12 months. In 2004, Brazil introduced a second dose of the trivalent MMR vaccine administered at 4 years of age. In 2013, the quadrivalent measles, mumps, rubella, and varicella vaccine administered at 15 months of age became the recommended second dose for measles-containing vaccines [3].

Although Brazil has reported routine childhood vaccination coverage rates above 95% since the 1990s [2], coverage rates have declined since 2015 [2–4], with downward trends exacerbated in 2020 likely due to the COVID-19 pandemic [5]. At the national level in Brazil, the MMR first dose vaccine coverage decreased from ≥95% (i.e., the MMR vaccine coverage target recommended by the Brazilian Ministry of Health [6]) in 2015 to <80% by 2020 [7]. Heterogeneity in MMR vaccine coverage between the five regions of Brazil also remains a challenge [2, 8, 9]. Whereas the mean MMR first dose vaccine coverage declined by 17% between 2015 to 2020 in the more deprived North and Northeast regions to 68% and 78% respectively, coverage in the wealthier South region dropped by only 11% to a coverage of 85% in 2020 [7]. A time-trend ecological analysis between 2006 and 2016 [9] also highlighted high-risk clusters in the North and Northeast states of Pará, Maranhão, and Bahia, where the proportion of children who received the MMR vaccine declined at a faster rate per year than in the rest of Brazil. Although Brazil was designated as measles-free in 2016, a re-emergence of measles occurred in 2018 with 10,346 cases reported, mainly in the Northern region of Brazil [2, 10, 11]. In 2019, an epidemic of measles caused 20,901 cases across 23 of the 26 Brazilian states—far exceeding the scale of outbreaks from the previous two decades [10, 11]. In 2020, a further 8,448 measles cases were reported [10]. Outbreaks of mumps have also occurred in Brazil, with the largest recent epidemic occurring in 2016 and affecting nine states, mainly from the South and Southeast regions [12]. Though no local transmission of rubella has been reported in Brazil since 2009, an imported case with no secondary transmission was reported in 2014, and the Brazilian Ministry of Health remains vigilant [13].

Despite improvements over the last three decades, Brazil continues to be challenged by a high degree of economic inequality and stark disparities in social and health conditions [14]. Since 2014, health inequalities have expanded in Brazil, while the nation has experienced a severe economic recession, a political crisis including the removal of a sitting president, a series of newly introduced austerity policies [15, 16], and COVID-19-related disruptions. Nevertheless, the association between declining rates of routine childhood vaccination coverage and community-level deprivation (i.e., the lack of basic material necessities, here reflecting low household incomes, illiteracy, and inadequate water/sanitation) in Brazil remains uncertain. To address this gap in knowledge, we conducted a nationwide ecological study within the period of 2006 to 2020 to investigate municipality-level MMR vaccine first dose coverage (i.e., as a continuous variable and as a binary variable for municipalities attaining the recommended 95% coverage target among children [6]) in relation to quintiles of municipality-level deprivation, measured by the Brazilian Deprivation Index (*Índice Brasileiro de Privação*, IBP) [17], and geographic regions.

## Materials and methods

### Study design and ethics statement

In this ecological study, we analysed publicly available nationwide data from Brazil on municipality-level MMR vaccine coverage and municipality-level deprivation measured by the IBP. As we exclusively used publicly available and aggregated data with no identifiable data at the individual level, the project was considered exempt from ethical approval in accordance with *Resolução N° 510* (7 April 2016) of the Brazilian Ethics System (*Sistema CEP-CONEP*).

### Vaccine coverage data

Vaccination coverage levels for the first dose of the MMR vaccine by municipality and year, from 2006 to 2020 inclusively, were obtained from the Brazilian Ministry of Health’s Unified Health System data registry (*Departamento de Informática do Sistema Único de Saúde*, DATASUS) [18]. Vaccine coverage levels as a percentage were based on data routinely collected by the National Immunization Program Information System (*Sistema de Informações do Programa Nacional de Imunizações*, SI-PNI) and administratively calculated at the municipality-level from the number of first doses of MMR vaccine administered divided by the target population (i.e., based on the number of live-born children in the prior year registered to the compulsory Live Birth Information System (*Sistema de Informações sobre Nascidos Vivos*, SINASC)) [19]. Using this approach, vaccine coverage in a given municipality and year may exceed 100%. Coverage levels exceeding 200% were excluded from analyses when the variable coverage was treated as a continuous variable. Hence, in the investigations of temporal differences of MMR coverage and for the linear association between deprivation and MMR, we excluded 1341 out of the 83,475 available vaccine coverage observations (1.61%) for which vaccine coverage was reported to exceed 200%. In a sensitivity analysis, we also investigated excluding observations with coverage levels exceeding 150%. MMR vaccine coverage levels were available in 5570 municipalities. The five municipalities (0.1%) that did not exist during the 2010 census, and therefore had missing data on the IBP, were excluded from all analyses.

### Deprivation data

The IBP is a small area deprivation index for Brazil, developed in 2020 based on 2010 (i.e., the most recently collected) population census data, which covers an estimated 99.7% of the population [20]. The IBP is a composite index synthesizing three census tract-level variables: low household income (i.e., the percentage of households with a per capita income of ≤1/2 minimum wage), illiteracy (i.e., the percentage of people aged seven years and above who are not literate), and inadequate water/sanitation (i.e., the mean percentage of people experiencing inadequate or no access to: toilet and bath/shower, sewage, water, and/or garbage collection). Population-weighted quintiles of the IBP are available in the source dataset and have been calculated such that each quintile includes a different number of municipalities but represents approximately 20% of the Brazilian population from the 2010 census. Of note, the population sizes of the 5565 municipalities recorded in the 2010 census ranged from 805 to more than 11.2 million residents; whereas 5% of municipalities had more than 1 million inhabitants, the majority had a population size ≤20,000 [17]. The IBP quintiles have been ordered from least (quintile 1) to most deprived (quintile 5). The IBP data were obtained from the Oswaldo Cruz Foundation’s Centre for Data and Knowledge Integration for Health (*Centro de Integração de Dados e Conhecimentos para Saúde*, CIDACS/Fiocruz, Salvador, Bahia, Brazil) [21].

### Statistical analysis

To investigate temporal patterns from 2006 to 2020, we first plotted the annual mean coverage of the first dose of the MMR vaccine by deprivation quintile, overall in Brazil, and within each of the five Brazilian regions. Quantitative estimates of the changes in MMR first dose vaccine coverage levels between 2006 and 2020 by deprivation quintiles and geographic regions were calculated using multilevel mixed-effects linear regressions with state-specific random effects (i.e., 5565 municipalities within 27 states). We then stratified the data and repeated the analyses in the periods of 2006–2013, 2014–2019, and 2019–2020 to better understand the longitudinal trends during the socioeconomic crisis in Brazil, beginning in 2014, and the COVID-19 epidemic, beginning in 2020. Of note, the year 2019 has been included within two subgroup analyses to allow more meaningful investigations of the changes occurring near the beginning of the COVID-19 epidemic in Brazil. We conducted two sensitivity analyses. First, we plotted the annual mean coverage of the second dose of the trivalent vaccine by deprivation quintile and region between 2013 and 2020 (i.e., the period with available MMR second dose vaccine coverage data). Second, we investigated, using multilevel mixed-effects linear regressions, the quantitative estimates of the changes in MMR first dose vaccine coverage levels by deprivation quintiles and geographic regions keeping observations with coverage levels lower than 150%.

To investigate patterns in the attainment of the 95% MMR vaccine coverage target, we first calculated the proportions of municipalities achieving the target in 2006 (i.e., baseline), 2013 (i.e., before the socioeconomic crisis), 2019 (i.e., after the socioeconomic crisis and before COVID-19), and 2020 (i.e., after COVID-19) and evaluated the differences in proportions between 2013 and 2019 as well as 2019 and 2020 using McNemar’s Chi-squared tests. We then mapped municipalities reaching 95% coverage by quintiles of the IBP in 2006, 2013, 2019, and 2020 using ggplot2 and geobr, an official spatial dataset of Brazil, R packages [22]. All analyses were performed using Stata, version 16.1, and R, version 4.0.3.

## Results

We analysed MMR first dose vaccine coverage from 5565 municipalities (99.9% of Brazilian municipalities) from 2006 to 2020. Overall, nearly half of the municipalities (46.1%) were in the most deprived fifth quintile, whereas only 4.0% were in the least deprived first quintile. Of note, none of the municipalities in the North and Northeast regions belonged to the first or second quintiles of IBP. The Central-West region also had no municipalities in the first quintile of deprivation. The South and Southeast regions had a more balanced distribution across the deprivation quintiles (S1 Table).

**Fig 1:**
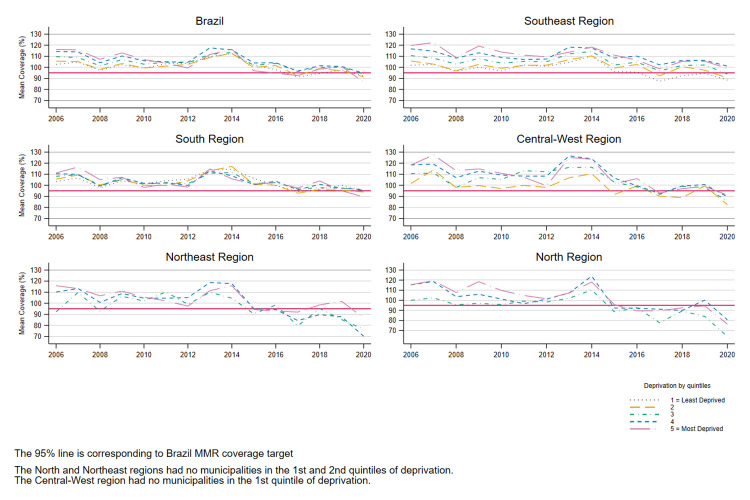
Temporal patterns of MMR first dose vaccine coverage between 2006 and 2020 by quintile of deprivation and region in Brazil. Note: Municipalities per region in 2020: Southeast n = 1654; South n = 1175; Central-West n = 463; Northeast n = 1783; North n = 449.

Between 2006 and 2015, MMR vaccine coverage in Brazil remained consistently above 95% (Fig 1). After 2015, mean coverage decreased in all regions—dropping below 95% coverage in some regions (North and Northeast)—and across all quintiles of the IBP. Before 2011, the lowest coverage levels were observed in the least deprived first and second quintiles, while the highest coverage was in the most deprived fourth and fifth quintiles of deprivation; after 2011, the differences across the quintiles decreased (Fig 1). In the sensitivity analysis, the mean coverage of the second dose of the MMR vaccine in Brazil between 2013 and 2020 was observed to be below the 95% coverage target with non-linear temporal patterns across the quintiles of deprivation (S1 Fig).

**Fig 2:**
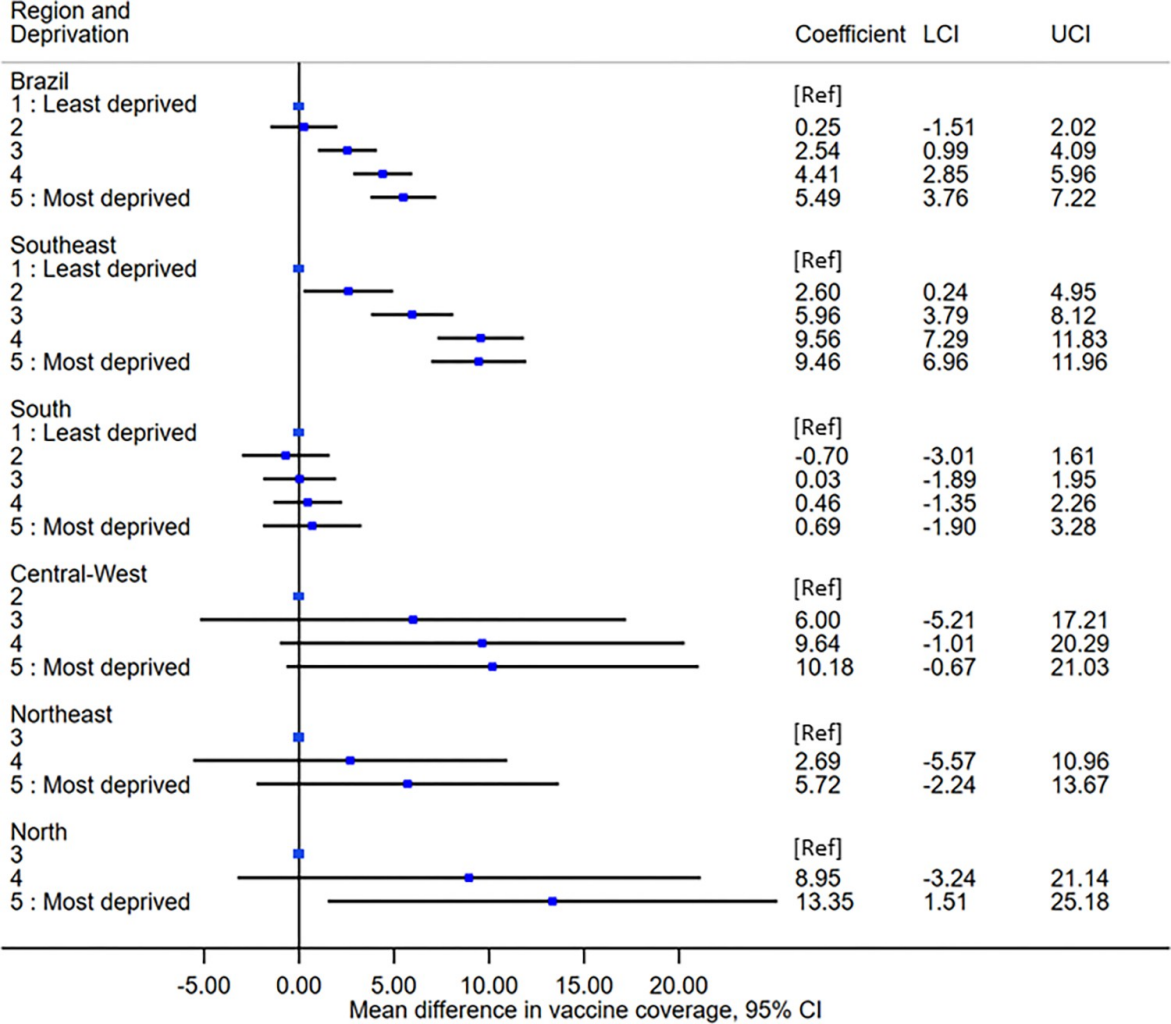
Mean differences, as percentages, in MMR first dose vaccine coverage between 2006 and 2020 by quintile of deprivation and region in Brazil, from multilevel mixed effects linear regressions, adjusted for year with state-specific random effects (5565 municipalities within 27 states). (Abbreviations: CI, confidence interval; LCI, lower confidence interval; UCI, upper confidence interval).

When analysing the association between MMR coverage and deprivation adjusted for year, there appeared to be a gradient between the deprivation quintiles and the MMR vaccine coverage overall (Fig 2). Between 2006 and 2020, municipalities in the most deprived fifth quintile had, on average, 5.49% (95% CI: 3.76 to 7.22%) higher MMR vaccine coverage than municipalities in the least deprived first quintile. Similar patterns were observed in all regions except for the South, in which there were no substantive differences in coverage across the deprivation quintiles over time. In the sensitivity analysis excluding observations with vaccine coverage exceeding 150%, we observed a similar, although less visible, gradient between the quintiles of deprivation, except for the Central-West region where no pattern was observed (S2 Fig).

The mean municipality-level MMR vaccine coverage in Brazil decreased, on average, by 1.22% per year (95% CI: 1.18 to 1.26%) between 2006 and 2020. Vaccine coverage declined, on average by 0.78% per year (95% CI: 0.69 to 0.86%) between 2006 and 2013 and subsequently 2.33% per year (95% CI: 2.16 to 2.49%) between 2014 and 2019 (Table 1). The annual declines in coverage between 2006 and 2020 appeared to be associated with the quintile of deprivation, such that the steepest declines were observed in the most deprived fifth quintile with an annual decrease of 1.64% (95% CI: 1.58 to 1.70%), and the least steep declines were observed in the least deprived first quintile with an annual decrease of 0.61% (95% CI: 0.47 to 0.75%) (Table 1). This gradient was observed in the periods of 2006–2013 and 2019–2020, but not in the period 2014–2019 when similar decreases in coverage were observed across all quintiles of deprivation. Between 2019 and 2020, the decline of coverage was steeper in the fifth quintile with a decrease in mean coverage of 14.10% in a year (95% CI: 12.92 to 15. 28) while in the first quintile the decrease was of 5.31% (95%CI: 1.90 to 8.72) (Table 1).

**Table 1:** Mean annual change, as percentages, in MMR first dose vaccine coverage by quintile of deprivation in Brazil, from multilevel mixed effects linear regressions, adjusted for year with state-specific random effects (5565 municipalities within 27 states).

Year	Quintile	Mean annual change in vaccine coverage (%)		
		Coefficient	95% CI	
2006–2020	**Overall**	**-1.22**	**-1.26**	**-1.18**
	1 (Least deprived)	-0.61	-0.75	-0.47
	2	-0.63	-0.75	-0.50
	3	-0.74	-0.83	-0.64
	4	-1.01	-1.08	-0.94
	5 (Most deprived)	-1.64	-1.70	-1.58
2006–2013 Before period of economic and political instability	**Overall**	**-0.78**	**-0.86**	**-0.69**
	1 (Least deprived)	0.68	0.39	0.97
	2	0.19	-0.07	0.44
	3	-0.01	-0.23	0.20
	4	-0.31	-0.48	-0.13
	5 (Most deprived)	-1.59	-1.72	-1.46
2014–2019* During period of economic and political instability	**Overall**	**-2.33**	**-2.49**	**-2.16**
	1 (Least deprived)	-2.92	-3.52	-2.32
	2	-2.58	-3.12	-2.04
	3	-2.24	-2.64	-1.85
	4	-2.56	-2.87	-2.25
	5 (Most deprived)	-2.12	-2.37	-1.87
2019*-2020 During onset of COVID-19 pandemic	**Overall**	**-9.75**	**-10.53**	**-8.97**
	1 (Least deprived)	-5.31	-8.72	-1.91
	2	-5.48	-7.97	-3.00
	3	-6.27	-8.20	-4.33
	4	-6.09	-7.54	-4.64
	5 (Most deprived)	-14.10	-15.28	-12.92

(Abbreviations: CI, confidence interval). *The year 2019 was included in both analyses.

By evaluating changes in the mean municipality-level MMR vaccine coverage in Brazil per geographical region, the average annual decreases between 2006 and 2020 ranged between 0.71% (95% CI: 0.63 to 0.79%) per year in the South region to 2.37% per year (95% CI: 2.23 to 2.51%) in the North region (Table 2). Between 2014 and 2019, the steepest drop in coverage was observed in the Central-West and North regions. Between 2019 and 2020, the North, Northeast, and Central-West regions had the largest decreases in mean coverage, adjusted for the deprivation quintile (Table 2).

**Table 2:** Mean annual change, as percentages, in MMR first dose vaccine coverage by region in Brazil, from multilevel mixed effects linear regressions, adjusted for year with state-specific random effects (5565 municipalities within 27 states).

Year	Region	Mean annual change in vaccine coverage (%)		
		Coefficient	95% CI	
2006–2020	**Brazil**	**-1.22**	**-1.26**	**-1.18**
	Southeast	-0.79	-0.86	-0.72
	South	-0.71	-0 .79	-0.63
	Central-West	-1.64	-1.79	-1.49
	Northeast	-1.55	-1.62	-1.48
	North	-2.37	-2.51	-2.23
2006–2013 Before period of economic and political instability	**Brazil**	**-0.78**	**-0.86**	**-0.69**
	Southeast	-0.30	-0.45	-0.15
	South	-0.17	-0.36	0.02
	Central-West	-0.15	-0.49	0.19
	Northeast	-1.48	-1.63	-1.33
	North	-2.01	-2.34	-1.68
2014–2019* During period of economic and political instability	**Brazil**	**-2.33**	**-2.49**	**-2.17**
	Southeast	-2.14	-2.42	-1.85
	South	-2.26	-2.59	-1.92
	Central-West	-4.04	-4.65	-3.44
	Northeast	-1.79	-2.09	-1.49
	North	-3.61	-4.15	-3.08
2019*-2020 During onset of COVID-19 pandemic	**Brazil**	**-9.75**	**-10.53**	**-8.97**
	Southeast	-6.66	-8.06	-5.26
	South	-2 .12	-3.74	-0.49
	Central-West	-10.65	-13.14	-8.17
	Northeast	-15.28	-16.69	-13.88
	North	-18.34	-20.81	-15.87

(Abbreviations: CI, confidence interval). *The year 2019 was included in both analyses.

**Fig 3:**
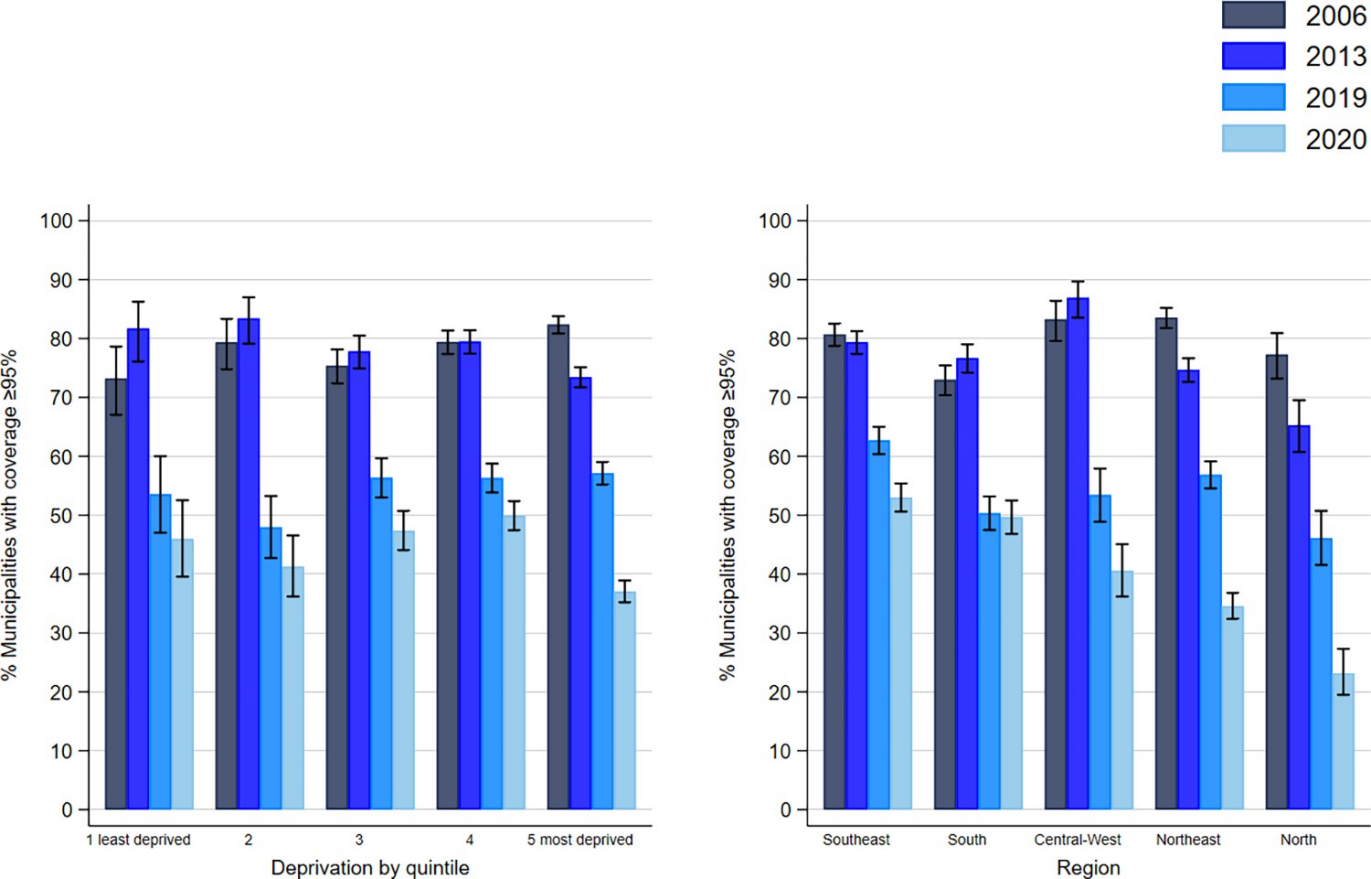
Longitudinal patterns for the percentages (with 95% confidence intervals) of municipalities with ≥ 95% of MMR first dose vaccine coverage by i) quintile of deprivation and ii) region in Brazil. Note: Municipalities included in the analysis: N = 5565. Municipalities per region: Southeast n = 1668; South n = 1188; Central-West n = 466; Northeast n = 1794; North n = 449. Municipalities per deprivation level: first quintile n = 224, second quintile n = 344, third quintile n = 857, fourth quintile n = 1575, fifth quintile n = 2565.

**Fig 4:**
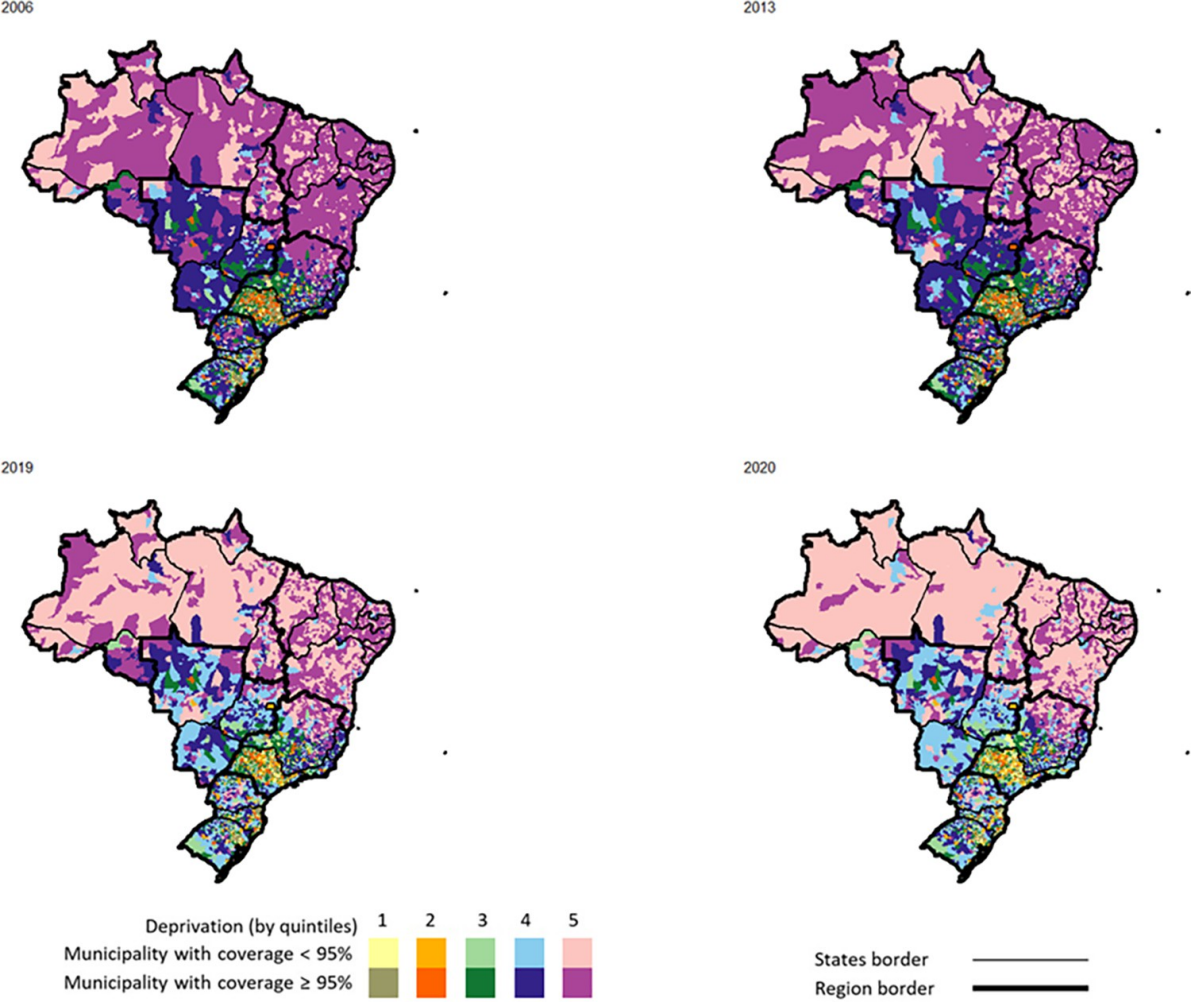
Spatial distribution of municipalities reaching 95% of MMR first dose vaccine coverage in relation to the deprivation level in Brazil. Note: Municipalities included in the analysis: N = 5565. Municipalities per region: Southeast n = 1668; South n = 1188; Central-West n = 466; Northeast n = 1794; North n = 449. Municipalities per deprivation level: first quintile n = 224, second quintile n = 344, third quintile n = 857, fourth quintile n = 1575, fifth quintile n = 2565. Source of the basemap shapefile available on https://github.com/ipeaGIT/geobr.

Comparing the years 2013 and 2019, all five IBP deprivation quintiles had lower percentages of municipalities reaching the 95% coverage target for the first dose MMR vaccine (p < 0.001 for all, McNemar’s Chi-squared test). The percentage of municipalities reaching the target dropped by 28.1% during that period in the least deprived first quintile, 35.5% in the second quintile, 21.5% in the third quintile, 23.2% in the fourth quintile, and 16.3% in the fifth more deprived quintile (Fig 3). Similarly, comparing 2013 and 2019, all regions had a lower proportion of municipalities reaching the 95% coverage target for the MMR vaccine (p<0.001 for all, McNemar’s Chi-squared test) (Fig 3). Comparing 2019 and 2020, all deprivation quintiles, except for the least deprived first quintile (p = 0.05), had a lower proportion of municipalities reaching the 95% target (p<0.001 for the third, fourth and fifth quintiles, and p = 0.03 for the second quintile, McNemar’s Chi-squared test); the largest drop between 2019 and 2020 was observed in the most deprived fifth quintile, where 20.1% of municipalities fell below the target in a year, while smaller but substantial differences of 7.6%, 6.7%, 8.9%, and 6.4% were respectively observed in the first, second, third, and fourth quintiles (Fig 3). Similarly, all regions, except for the South region, had a lower proportion of municipalities reaching ≥95% coverage between 2019 and 2020 (p<0.001 for all, McNemar’s Chi-squared test), with a difference of 22.9% in the North region, 22.3% in Northeast region, 12.9% in Central-West region, 9.7% in the Southeast region, and 0.7% in the South region (Fig 3). Across all deprivation quintiles and regions, except for the Southeast region, the 95% coverage target was attained by less than 60% of municipalities in 2019 and by less than 50% in 2020 (Fig 3). When plotted onto the map of Brazil, the drop in coverage between 2006, 2013, 2019 and 2020 can be observed to span all regions and deprivation levels (Fig 4).

## Discussion

In this study, we have analysed the ecological association between municipality-level MMR first dose vaccine coverage and deprivation in Brazil from 2006 to 2020. The more deprived municipalities had, on average, higher coverage levels between 2006 and 2020. However, the mean municipality-level MMR vaccine coverage declined on average by more than 1% per year during this period, with the most pronounced declines observed in the more deprived quintiles and in the more deprived North, Northeast and Central-West regions. Similarly, between 2019 and 2020 (i.e., the beginning of the COVID-19 pandemic period), our analysis found that the most deprived fifth quintile of municipalities, as well as those in the North and Northeast regions, experienced the largest drop both in mean MMR vaccine coverage, as a continuous variable, and in the percentage of municipalities reaching the 95% coverage target. As of 2020, in all regions except the Southeast, less than 50% of Brazilian municipalities had reached the 95% MMR vaccine coverage target recommended by the Brazilian government [6].

Our longitudinal analyses align with the growing body of evidence demonstrating declining childhood immunization coverage in Brazil, which has been hypothesized to be attributable, in part, to growing vaccine hesitancy [2–4, 7–9, 23]. Despite the overall downwards trends, we did observe higher coverage in 2013 and 2014 that might be explained by behavioural changes due to the 2013–2015 measles epidemics [2] or by the shift in data collection methods (i.e., from an offline monthly reporting system to a real-time electronic immunization registry [9]) that occurred in 2013.

In analyses stratified by time period, we found steeper declines in vaccine coverage in Brazil during the period of 2014–2019 and 2019–2020, as compared to 2006–2013, across all regions and deprivation quintiles. Although the specific causes remain to be determined, the socioeconomic crisis in Brazil, beginning in 2014, coupled with the austerity policies beginning in 2016, have likely played a role in increasing health inequalities and contributing to under-vaccination [15, 16, 24]. Notably, the reduced financing of the healthcare system, has occasionally led, among other consequences, to shortages of vaccines [3, 9, 23]. Whereas the declines in MMR first dose vaccine coverage from 2014 onwards were similar across deprivation quintiles and regions, our results show deprivation-related and regional differences in MMR vaccine coverage between 2019 and 2020, with the largest drops occurring in municipalities of the most deprived fifth quintile and the North and Northeast regions. These findings, which are of significant public health concern, are similar to those of a recent study using individual-level data from a nationwide survey showing that disruptions related to the COVID-19 pandemic were associated with reduced uptake of childhood vaccinations in general, with children from poor families and from the least developed regions of Brazil more affected [5]. The drop in the coverage of the first dose of a measles-containing vaccine (MCV1) has been reported to continue during the COVID-19 pandemic, and 2021 had the lowest coverage in MCV1 since 2008 worldwide, with Brazil identified as one of the top ten countries in the world with the highest proportion of infants who did not receive a MCV1 [25]. Understanding the specific kinds of causal mechanisms (e.g., supply chain issues, reduced healthcare contact) underlying the different patterns of decline during these two periods may help to build resilience in the health service. To further bolster control of measles, mumps, and rubella, a few approaches have been suggested in a recent literature review, including educating the population on disease severity and the value of vaccination, improving surveillance systems to facilitate rapid responses to decreases in coverage, improving outbreak preparedness (i.e., plans that take into account delays in the release of vaccines, including vaccination of healthcare workers and considering an early dose for infants from 6 months of age), identifying and targeting unvaccinated or under-vaccinated individuals for interventions, and strengthening the system weaknesses (e.g., health infrastructure access and management, surveillance system, vaccine supply) [26].

The finding of lower MMR first dose vaccine coverage among the least deprived municipalities shares some similarities with the results of a number of individual-level studies from 2008 and 2015, which have shown lower rates of achieving complete vaccination schedules by 12 or 18 months of age among families in Brazil with higher socioeconomic position [27, 28]. Our results may be partially explained by the social benefits that are offered to the most deprived families. For example, the *Bolsa Família* conditional cash transfer program for low-income families included vaccination of children as a conditionality for receiving benefit and has been associated with increased odds for vaccination among children under seven in a 2010 household survey of a *favela* community within the Northeastern city of Salvador [29]. Similarly, a 2000–2002 randomized intervention evaluation in rural Nicaragua reported that the national conditional cash-transfer pilot program led to increases in vaccination, especially in children living far away from a health facility or whose mothers were less educated [30]. Of note, as the *Bolsa Família* program was ended in 2021 and recently resumed in 2023 [31], further research, such as interrupted time series analyses, is warranted to evaluate the impact of the program on vaccine coverage rates. Individual-level studies assessing vaccine uptake will be particularly valuable to control for other confounding factors, such as socioeconomic position, as other individual-level studies from 2005 and 2010 have reported incomplete vaccine coverage among children from families with lower socioeconomic position indicators [32, 33]. Additionally, further work is needed to investigate other dimensions of social determinants, such as municipality-level income segregation or racial disparities.

The observed regional differences in vaccine coverage across Brazil are consistent with prior research. Previous studies have reported lower vaccine coverage, larger gaps in coverage decrease over time, and more missed vaccine doses [4–9] in the most deprived North and Northeast regions [34]. While decentralization of immunization organisation to the municipality-level under PNI has contributed to reduced regional inequities [3], smaller municipalities may face persistent challenges, such as staff shortages, with higher turnover and lack of training [3, 35]. The Pan American Health Organization recommends that all countries meet the goal of achieving ≥95% of coverage for each dose of the MMR vaccine in at least 80% of municipalities [36]. In Brazil, the Unified Health System has set a target for ‘homogeneity’ of 70% of municipalities reaching more than 95% of MMR coverage at 12 months of age [8]. In our study, across all deprivation quintiles and regions, the 70% homogeneity target was achieved in 2006 and 2013 (except for the North in 2013) but not in 2019 or 2020. These widespread pockets of low MMR vaccine coverage may enable more frequent outbreaks in the future.

This longitudinal analysis provides important insights into the association between the coverage of the first dose of the MMR vaccine and municipality-level deprivation in a large heterogeneous middle-income country. However, there are limitations. First, inherent to this study’s design, ecological fallacy is a major concern for interpretation, and it is important to emphasize that the observed associations between MMR coverage and deprivation at the municipality-level may not be replicable at the individual-level. Further research integrating data collected at the municipality-, household-, and individual-levels is warranted to understand which aspects of deprivation are the most important risk factors for missing, incomplete, or delayed MMR vaccination [37]. Second, residual confounding may also be present from unmeasured factors (e.g., accessibility of healthcare facilities) at the municipality level. Third, misclassification in both the IBP and the vaccine coverage data may have attenuated the effect estimates. As the IBP was based on the most recent 2010 census while the vaccine coverage data spanned the period of 2006 to 2020, it is likely that the relative deprivation of specific municipalities will have varied over time. In the SI-PNI dataset, the estimation of MMR coverage assumed that the vaccine was distributed to the target population (i.e., children born in the municipality the year prior) but the denominator might not be accurate. It does not consider, for example, migrant children or infant mortality over the first year of life. Arroyo and colleagues also hypothesized that children might be born and live in different municipalities and that municipalities offering easier access to vaccination rooms may administer higher numbers of doses, including to children from neighbouring municipalities [9]. This routinely collected vaccine coverage data were the best that were available, as they cover all of Brazil identically; however, the administrative method of calculating coverage may have led to an over-estimation of coverage in some municipalities, as reflected in the reported mean coverage levels above 100%. Although we acknowledge that the recommended 95% vaccine coverage target may not be accurate in this situation, our longitudinal analysis showed temporal patterns and the drop from mean coverage higher than 100% to less than 95% is concerning. Finally, as the two datasets containing information on the coverage of the MMR vaccine second dose (i.e., the trivalent and the quadrivalent vaccines) did not cover our study period entirely and used a different denominator for the calculation of the coverage [19], this study opted to focus on the coverage of the first rather than the second dose of MMR, although we recognize the importance of the second dose for achieving population-level immunity.

## Conclusion

The findings from this study highlight a widespread decrease in MMR vaccine first dose coverage, but with the most striking decreases seen in the most deprived municipalities and the poorest regions in Brazil. Our findings reaffirm regional socioeconomic and health disparities, with the most deprived North and Northeast regions experiencing the largest inter-annual decreases in MMR vaccine coverage. These findings also call attention to the fact that the most deprived municipalities have experienced the most rapid decreases in vaccine coverage over time, as well as the greatest drops in coverage levels during the beginning of the COVID-19 pandemic. To promote vaccine equity and prevent future outbreaks, further research is urgently needed to understand the causal mechanisms underlying the observed associations between community-level MMR vaccine coverage and deprivation in Brazil.

## Supporting information

S1 Table Distribution of municipalities by deprivation levels and regions. (DOCX)

S1 Fig Temporal pattern of MMR second dose vaccine coverage by quintiles of deprivation in Brazil and stratified across all 5 regions, 2013–2020. (TIF)

S2 Fig Mean differences, as percentages, in MMR first dose vaccine coverage, keeping observations with coverage ≤150%, between 2006 and 2020 by quintile of deprivation and region in Brazil, from multilevel mixed effects linear regressions, adjusted for year with state-specific random effects (5565 municipalities within 27 states). (Abbreviations: CI, confidence interval; LCI, lower confidence interval; UCI, upper confidence interval).
(TIF)
