# Supplementary material for: Municipality-level measles, mumps, and rubella (MMR) vaccine coverage and deprivation in Brazil: A nationwide ecological study, 2006 to 2020
Source: PLOS Glob Public Health. 2023 Aug 1;3(8):e0002027. doi: 10.1371/journal.pgph.0002027 (PMC10393142; doi:10.1371/journal.pgph.0002027)
Supplement: S1 Table — (DOCX) [file pgph.0002027.s001.docx]

|  | | **Population-weighted deprivation quintiles** | | | | | |  |
| --- | --- | --- | --- | --- | --- | --- | --- | --- |
|  | **1 = Least deprived** | | **2** | **3** | **4** | **5 = Most deprived** | **Total** | |
| **Brazil** | 224 (4.03%) | | 344 (6.18%) | 857 (15.40%) | 1575 (28.30%) | 2565 (46.09%) | 5565 | |
| Central-West | 0 (0.00%) | | 5 (1.07%) | 40 (8.58%) | 327 (70.17%) | 94 (20.17%) | 466 | |
| Northeast | 0 (0.00%) | | 0 (0.00%) | 7 (0.39%) | 80 (4.46%) | 1707 (95.15%) | 1794 | |
| North | 0 (0.00%) | | 0 (0.00%) | 4 (0.89%) | 52 (11.58%) | 393 (87.53%) | 449 | |
| Southeast | 101 (6.06%) | | 223 (13.37%) | 508 (30.46%) | 547 (32.79%) | 289 (17.33%) | 1668 | |
| South | 123 (10.35%) | | 116 (9.76%) | 298 (25.08%) | 569 (47.90%) | 82 (6.90%) | 1188 | |

**S1 Table. Distribution of municipalities by deprivation levels and regions**
