# Supplementary figures and images for: Municipality-level measles, mumps, and rubella (MMR) vaccine coverage and deprivation in Brazil: A nationwide ecological study, 2006 to 2020
Source: PLOS Glob Public Health. 2023 Aug 1;3(8):e0002027. doi: 10.1371/journal.pgph.0002027 (PMC10393142; doi:10.1371/journal.pgph.0002027)

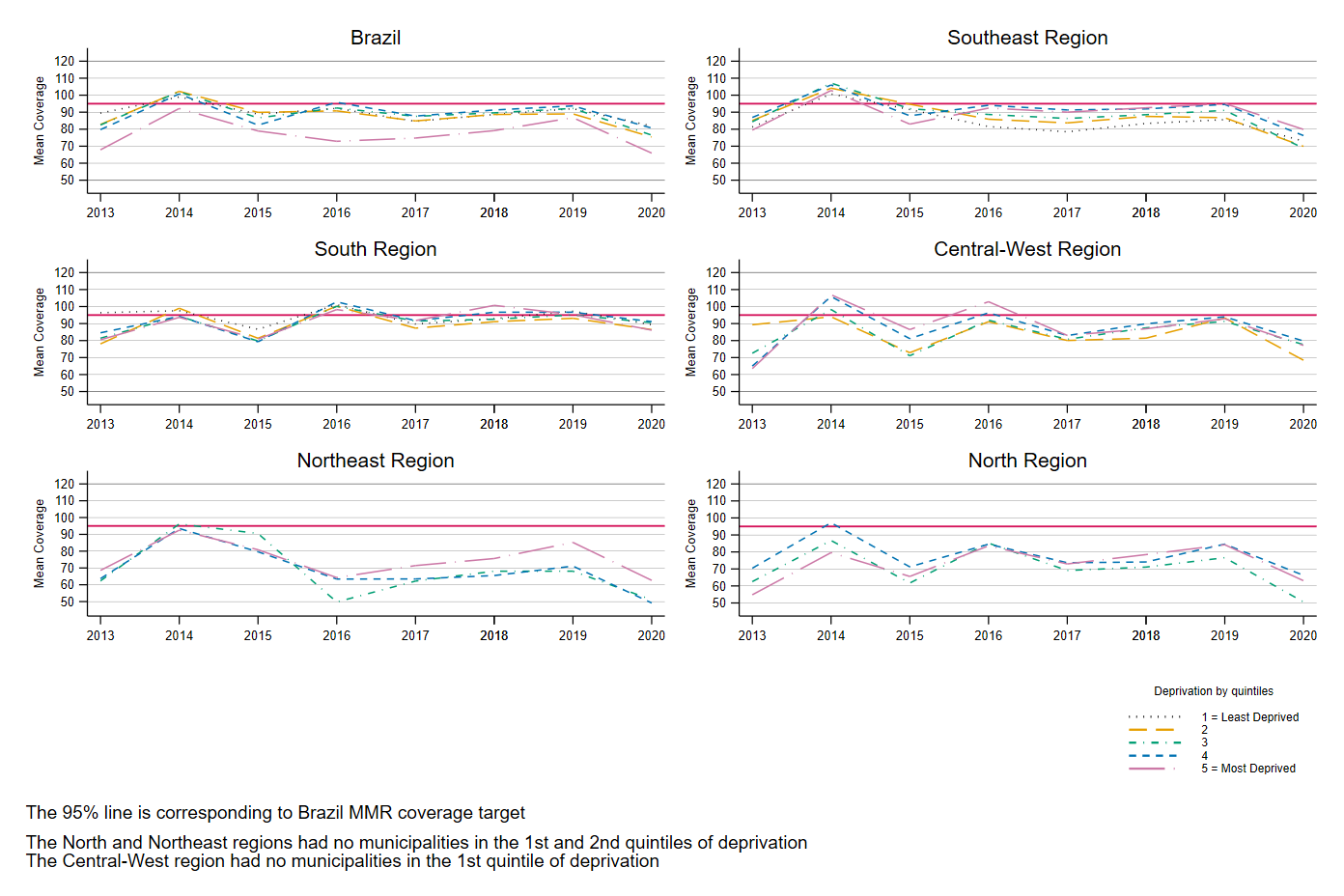

Supplement: S1 Fig — (TIF) [file pgph.0002027.s002.tif]

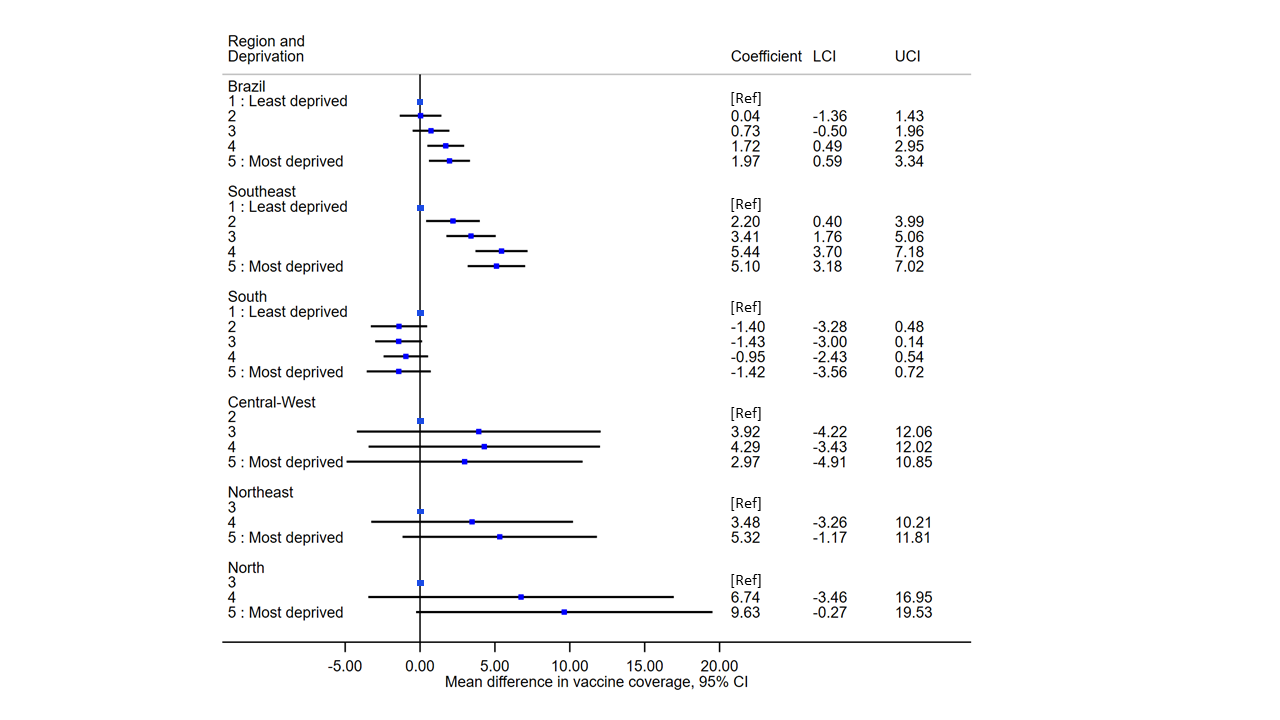

Supplement: S2 Fig — (Abbreviations: CI, confidence interval; LCI, lower confidence interval; UCI, upper confidence interval). (TIF) [file pgph.0002027.s003.tif]
